# In vitro effects of gamma-secretase inhibition in HPV-positive and HPV-negative head and neck squamous cell carcinoma

**DOI:** 10.1007/s10637-023-01334-x

**Published:** 2023-02-21

**Authors:** Sara Varatanovic, Tobias Maier, Sega Al-Gboore, Stefan Stoiber, Sam Augustine Kandathil, Clemens Quint, Charlotte Brennus, Gregor Heiduschka, Lorenz Kadletz-Wanke, Faris F. Brkic

**Affiliations:** 1 Department of Otorhinolaryngology, Head and Neck Surgery, Medical University of Vienna, Vienna, Austria; 2 Department of Pathology, Medical University of Vienna, Vienna, Austria; 3 Christian Doppler Laboratory for Applied Metabolomics, Medical University of Vienna, Vienna, Austria; 4 Division of Anatomy, Center for Anatomy and Cell Biology, Medical University of Vienna, Vienna, Austria

**Keywords:** Head and neck squamous cell carcinoma, Notch pathway, Gamma-secretase inhibitor

## Abstract

**Background:**

New chemotherapy agents are warranted for head and neck squamous cell carcinoma (HNSCC), particularly for incidence-rising HPV-positive tumors. Based on the evidence of Notch pathway involvement in cancer promotion and progression, we aimed to gain insights into the in vitro antineoplastic effects of gamma-secretase inhibition in HPV-positive and -negative HNSCC models.

**Methods:**

All in vitro experiments were conducted in two HPV-negative (Cal27 and FaDu) and one HPV-associated HNSCC cell line (SCC154). The influence of the gamma-secretase inhibitor PF03084014 (PF) on proliferation, migration, colony forming, and apoptosis was assessed.

**Results:**

We observed significant anti-proliferative, anti-migratory, anti-clonogenic, and pro-apoptotic effects in all three HNSCC cell lines. Furthermore, synergistic effects with concomitant radiation were observable in the proliferation assay. Interestingly, effects were slightly more potent in the HPV-positive cells.

**Conclusion:**

We provided novel insights into the potential therapeutic relevance of gamma-secretase inhibition in HNSCC cell lines in vitro. Therefore, PF may become a viable treatment option for patients with HNSCC, particularly for patients with HPV-induced malignancy. Indeed, further in vitro and in vivo experiments should be conducted to validate our results and decipher the mechanism behind the observed anti-neoplastic effects.

## Introduction

Head and neck squamous cell carcinoma (HNSCC) is the sixth most common malignancy worldwide and is associated with low survival rates [1]. Due to the reduced exposure to typical risk factors, such as alcohol and tobacco, a steady decline in HNSCC incidence has been observed in recent years. However, due to the global spread of human papillomavirus (HPV), the incidence of HPV-induced HNSCC is steadily rising. In particular, high-risk HPV types, particularly HPV 16, have been linked to the development of HNSCC [2].

HPV-related oncogenesis revolves primarily around the HPV-associated oncoproteins E6 and E7, which promote degradation of the tumor-suppressor genes TP53 and RB1 and modulate important cell cycle processes, resulting in tumor promotion and progression [3]. In contrast to HPV-negative tumors, HPV-positive HNSCC has shown better responses to chemotherapy and radiation therapy, [4] resulting in better survival rates [5–7].

HPV-positive tumors differ from HPV-negative disease with regards to genetic alternations. For example, FGFR2 and FGFR3 mutations are uniquely found in HPV-positive tumors [8]. Due to substantial clinical, molecular, and biological differences between HPV-positive and -negative HNSCC, these two malignancies are widely considered as two distinct cancer types [9].

Cisplatin is the most commonly used systemic agent treatment option for advanced HNSCC. However, the response to therapy remains highly variable among patients. One important limitation is the cisplatin resistance, which often associates with termination of treatment in HNSCC patients [10]. Furthermore, intravenous administration of cisplatin has been shown to increase initial tissue accumulation and plasma levels for an extended time periods and its traces are detectable years after treatment [11]. Its use is often hindered by adverse effects, such as nausea and vomiting, ototoxicity, fever, hyposthenia, altered sleep–wake cycle, and alterations in the liver, skin and respiratory system [12]. Other systemic therapy options include cetuximab and panitumumab. However, in spite of promising preliminary results, both agents showed no major survival improvements [13].

The Notch pathway is involved in cell processes such as cell proliferation, differentiation, and survival. It operates by transmitting extracellular information, depending on the cell–cell contact interactions, and plays a role in cell cycle regulation. It is often upregulated in cancer, and many Notch signaling alterations have been linked to oncogenesis [14, 15]. Preliminary results have shown the involvement of important Notch pathway proteins (Notch1 to Notch4) in HNSCC carcinogenesis [16]. Moreover, the Notch cascade has even been linked to cisplatin resistance [17]. One possibility of Notch pathway inhibition is the gamma-secretase, which is a protein complex that facilitates the cleavage of the intracellular Notch domain, therefore activating Notch signaling. Hence, targeting the Notch pathway via gamma-secretase inhibition may be a viable treatment option for cancer patients, particularly HNSCC [18].

As no substantial improvements in chemotherapy options for HNSCC in recent years, efforts need to be given to the identification of new therapeutic agents [11–13]. Therefore, the aim of the current study was to investigate potential anti-neoplastic effects of PF03084014 (PF), a gamma-secretase inhibitor (GSI), in HPV-positive and HPV-negative HNSCC cell lines and to gain insights into its therapeutic relevance in HNSCC. In particular, we assessed anti-proliferative, -migratory, -clonogenic, and pro-apoptotic properties of PF in HNSCC.

## Methods

### Cell culture

All experiments were conducted in three HNSCC cell lines, two HPV-negative (FaDu and Cal27, hypopharyngeal and tongue squamous cell carcinoma, respectively) and one HPV-positive cell line (SCC154, tongue squamous cell carcinoma) [19], which were purchased from the American Type Culture Collection (ATCC, Manassas, VA, USA). Cells were split every 5–8 days, depending on the seeding density. All cell lines were used only up to the 30th passage due to the risk of accumulating additional mutations. The cultivation was performed using Dulbecco’s modified eagle’s medium (DMEM), 1% Penicillin/Streptomycin (P/S), and 10% fetal calf serum (FBS), which were obtained from Gibco (Gibco, Thermo Fisher Scientific, Waltham, MA, USA). The incubation environment for the cells was 37 °C and 5% CO2 (Hera Cell 240, Heraeus Holding GmbH) cultivated in DMEM. Cells were split by removing the medium, washing the cells with Dulbecco’s Phosphate Buffered Saline (DPBS) (Gibco, Thermo Fischer Scientific, Waltham, Massachusetts, USA) and 0.05% trypsin – 0.53 mM EDTA solution (Sigma-Aldrich, St. Louis, Missouri, USA) to detach the cells. The inhibitor PF was acquired from Selleckchem (Houston, Texas, USA) [20]. In all experiments, three biological replicates were conducted.

### Cytotoxicity assay

In order to determine the cytototoxic effects of PF and calculate its half-maximal inhibitory concentration (IC50) value of PF, we performed a dose–response assay in 96-well plates (Sarstedt, Nurnbrecht, Germany) for each cell line. In particular, 5000 cells/well were seeded in 100 μl DMEM. After 24 h (h) of incubation, cells were exposed to gradually increasing concentrations of the inhibitor. The inhibitor was reconstituted in dimethyl sulfoxide (DMSO) (Sigma-Aldrich, St. Louis, Missouri, USA) and diluted in cell culture medium (2.5–40 μM for FaDu and Cal27, and 1.25–20 μM for SCC154 with five replicates per dose). Vehicle control was conducted using 0.1% DMSO. PF treatment was followed by irradiation with 2, 4, and 8 Gray (Gy) with the YXLON device (YXLON, International GmbH, Hamburg, Germany). After 72 h, the medium containing the inhibitor/vehicle control was removed and replaced with 100 μl of 56 μM resazurin (Sigma-Aldrich, St. Louis, Missouri, USA) solution. FaDu and Cal27 were then incubated for 90 min (min), while SCC154 were incubated for 180 min. Measurements were performed using the TECAN Spark reader (TECAN Spark, Tecan Group Ltd, Maennedorf, Switzerland).

### Migration assay

In order to assess the anti-migratory potential of the PF, we used 24-well plates (Greiner Bio-One, Frickenhausen, Germany) with Ibidi inserts (Greiner Bio-One, Frickenhausen, Germany) to create the initial gap. For this, 70,000 Cal27 and FaDu cells were seeded in Ibidi chambers (140,000/well) and 250,000 SCC154 cells/ibidi chamber in starvation medium (DMEM with 1% P/S and 1% FBS). Ibidi inserts were removed after reaching 100% cell confluency. Upon removal of the Ibidi inserts, wells were washed with DPBS and introduced with starving medium containing two different inhibitor concentrations (17 and 23 μM for Cal27 and FaDu, 10 and 20 μM for SCC154), as well as the vehicle control (0.1% DMSO). Visualizations using the TECAN Spark plate reader were utilized after 0, 24, and 48 h. Calculations and analysis of the obtained images were performed with ImageJ.

### Colony forming assay

Furthemore, to determine a single-cell’s ability to form colonies after treatment, we performed a clonogenic assay. In particular, 1000 SCC154 cells/well and 350 cells/well for Cal27 and FaDu were seeded in 12-well plates (Sarstedt, Nurnbrecht, Germany) containing 1 mL of medium. Treatment was performed 24 h after seeding (23 μM PF for Cal27 and FaDu and 20 μM for SCC154). After 72 h of treatment, cells were incubated for 10 (Cal27 and SCC154) and 14 days (FaDu). Subsequently, wells were washed with DPBS to remove cell debris, and the visualization was performed with the TECAN Spark plate reader.

### Apoptosis assay

Caspase 3/7 Glo Assay (Promega, Fitchburg, Wisconsin, USA) was conducted to assess the pro-apoptotic effects of the inhibitor. For Cal27 and FaDu, 7.500 cells/well and 15,000 SCC154 cells/well in 100 μL DMEM were seeded into 96-well plates (Sarstedt, Nurnbrecht, Germany). After 24 h, the medium was cells were treated. After 72 h, the medium was collected and mixed in a 1:1 ratio with Caspase 3/7 Glo reagent in three conditions: blank (media + reagent, no cells), tested sample (media containing inhibitor, treated cells + reagent), and negative control (media containing vehicle control + reagent and treated cells). We prepared the reagent according to the manufacturer’s instructions (Promega). Cells were incubated for 30 min and the luminescence was measured with the TECAN Spark plate reader.

### Statistical analysis

In order to compare differences in cell viability between different inhibitor concentrations, we conducted a two-way ANOVA analyses. On the other hand, differences in the migration, the colony formation, and the apoptosis assays were analyzed with a one-way ANOVA. For both, multiple comparison testings were performed on all possible pairwise means. We used GraphPad Prism (GraphPad Software, San Diego, California, USA) for graphical representation. All figures show mean values ± standard deviation (SD). Synergistic interactions between the inhibitor and radiation therapy were evaluated using the online tool Synergy Finder using a zero-interaction potency (ZIP) model.

## Results

### PF treatment leads to a reduction in cell viability

**Fig. 1:**
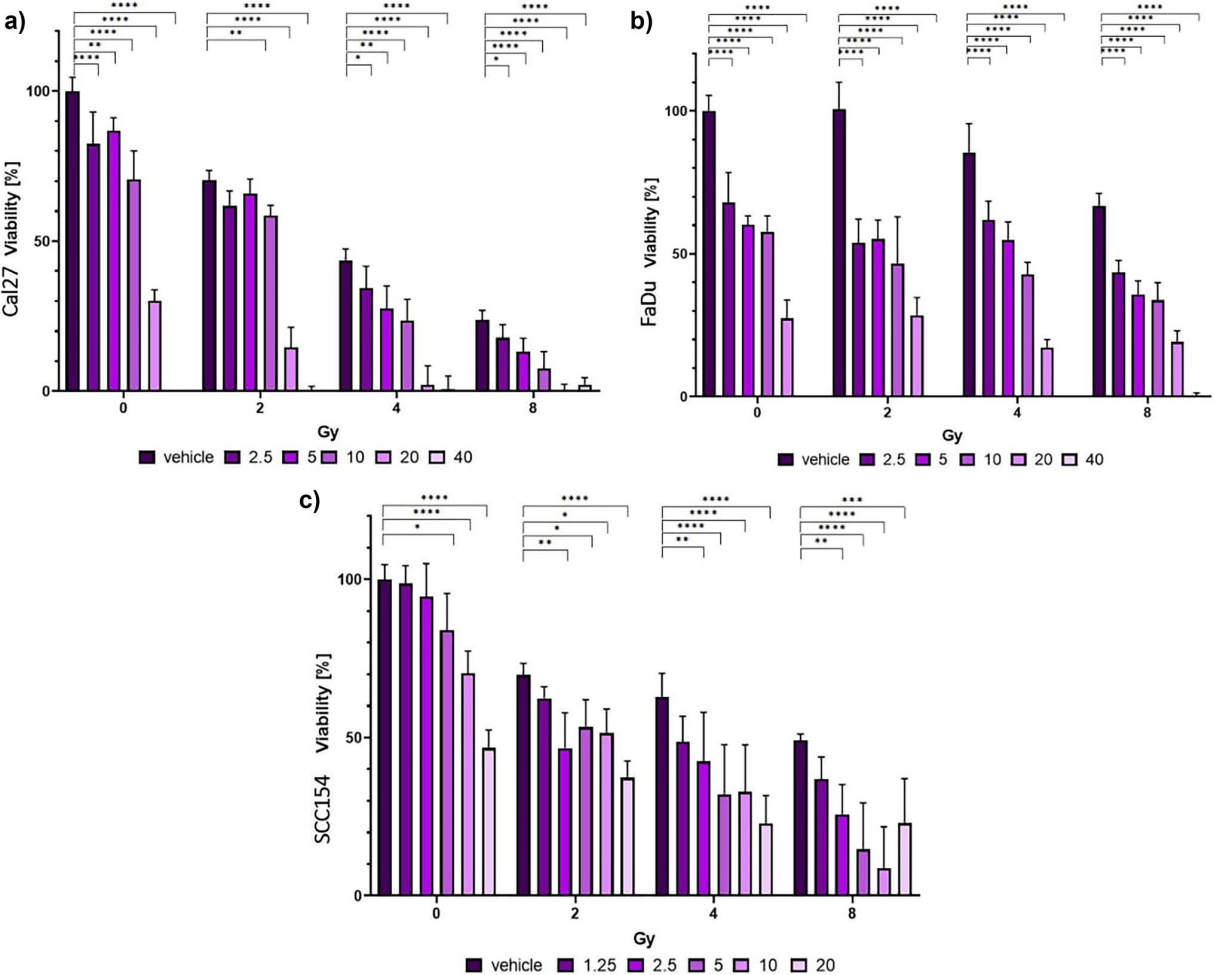
Dose–response assay results show decreased cell viability after treatment with PF. **a** Cal27 cell line treated with serial dilutions of PF (2.5–40 μM). Cytotoxic effects were observed with the IC50 value calculated at 23 μM. Significant differences in cell viability were observed through all inhibitor concentrations and in all radiation levels. **b** At the highest concentration, cell viability was successfully decreased to an almost non-viable level and the IC50 value was calculated as 23 μM for FaDu cells. **c** Treatment with PF showed the most potent anti-neoplastic effects in SCC154. Gy; Gray. Significant differences between controls and PF-treated cells are represented with asterisks (*; *p* < 0.05, **; *p* < 0.01, ***; *p* < 0.001, ****; *p* < 0.0001)

**Fig. 2:**
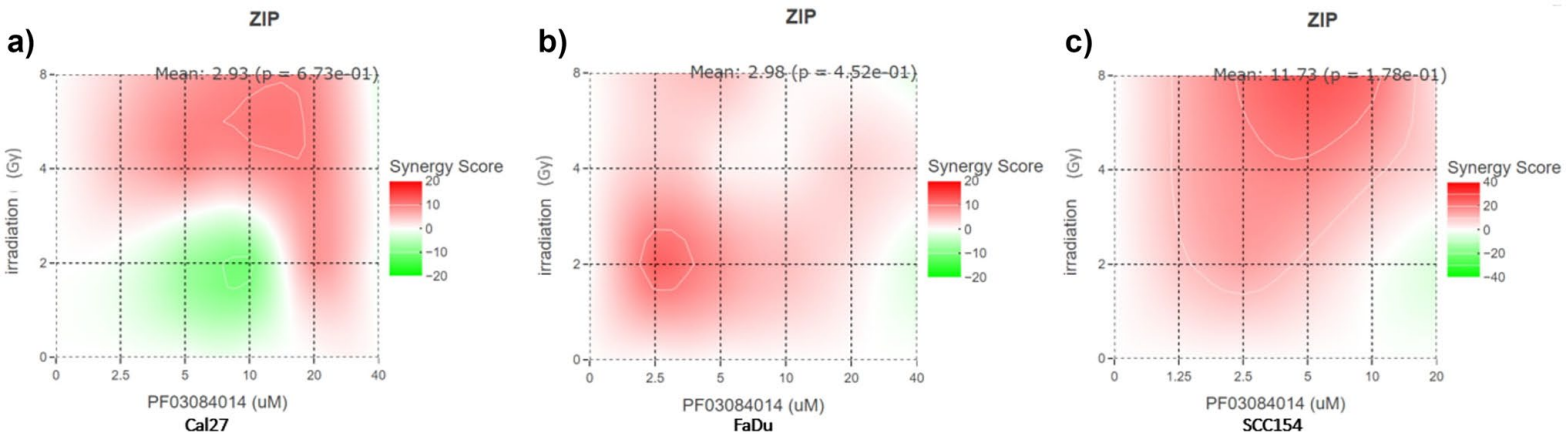
Synergistical effects of PF inhibitor and irradiation treatment Cal27, FaDu and SCC154 cell line. **a** Cal27 cell line, treated with PF dilutions (2.5–40 μM) and 2-8 Gy irradiation. This cell line shows the strongest synergy signal at 4 Gy and 20 μM PF. **b** FaDu cell line, treated with PF dilutions (2.5–40 μM) and 2-8 Gy irradiation. Strongest irradiation signal is observed at 2 Gy irradiation and 2.5 μM PF. **c** SCC154 cell line, treated with PF dilutions (1.25–20 μM) and 2-8 Gy irradiation. Strongest signal of synergy observed at 8 Gy irradiation and 5 μM PF concentration. Figures obtained using synergyfinder.com

In order to assess the cytotoxicity of the PF in HNSCC, we performed the dose–response assay. For these, 5000 cells/well were seeded into 96-well plates with in 100 μl DMEM for each cell line. After 24 h of incubation, we treated cells with increasing concentrations of the inhibitor. We observed significant anti-proliferation effects of PF in all three cell lines (Fig. 1). In particular, the IC50 concentration for SCC154 was 20 μM. Meanwhile, it was 23 μM for both, the Cal27 and the FaDu cell line, indicating a slightly higher sensitivity of the HPV-positive cell line. Regarding synergy, cell viability data obtained from the dose–response assay was used to calculate synergy scores. Synergy reflects an excess response due to drug and radiation interactions. Importantly, scores higher than 10 are interpreted as synergistic interactions, scores between 10 to − 10 as additive effects, and scores under − 10 suggest antagonistic effects. Here, we observed mostly additive and synergistic effects of radiation and PF treatment in all three cell lines. Furthermore, the strongest synergy levels shown for SCC154 (Fig. 2).

### PF impairs the migratory potential of HNSCC cell lines

**Fig. 3:**
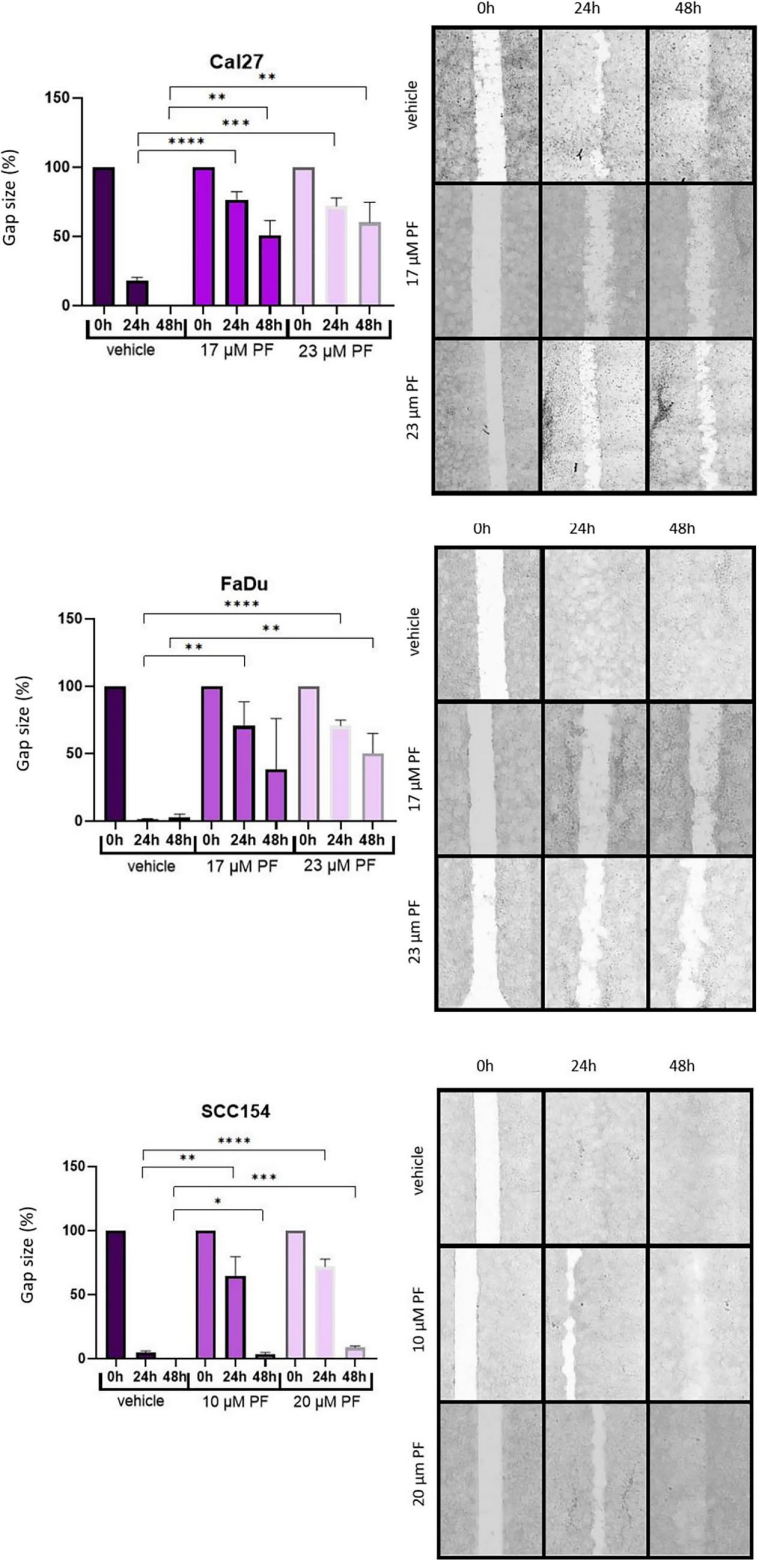
Anti-migratory effects of PF treatment on Cal27, FaDu and SCC154 cell lines. Subfigures depict corresponding representative migration gap closures. Significant differences between controls and PF-treated cells are represented with asterisks (*; *p* < 0.05, **; *p* < 0.01, ***; *p* < 0.001, ****; *p* < 0.0001)

We utilized a wound healing assay to assess the effects of PF on the migratory potential of three HNSCC cell lines and graphically depicted these results in Fig. 3, showing mean gap closure ± SD. As aforementioned, the corresponding IC50 values of PF were used for treatment. In particular, we used 24-well plates and the gap was created with Ibidi insets. In total, strong anti-migratory effects were observed in all three cell lines, particularly in FaDu and Cal27. Specifically for Cal27, treatment with 23 μM PF significantly reduced migration ability after 24 h and 48 h. Similarly, strong anti-migratory potential was shown in FaDu cells after treatment with 23 μM of PF and gap closure was significantly reduced at the 24 and 48 h time points (p < 0.0001 and p = 0.0255, respectively). A slightly less potent anti-migratory potential of PF was observed in SCC154. Still, the gap closure significantly decreased 24 h and 48 h after treatment with 20 μM of PF (p = 0.0118 and p = 0.0287, respectively). Furthermore, similar effects were observed in all cell lines after treatment with lower inhibitor concentrations.

### PF decreases colony formation in HPV-negative and -positive HNSCC cell lines

**Fig. 4:**
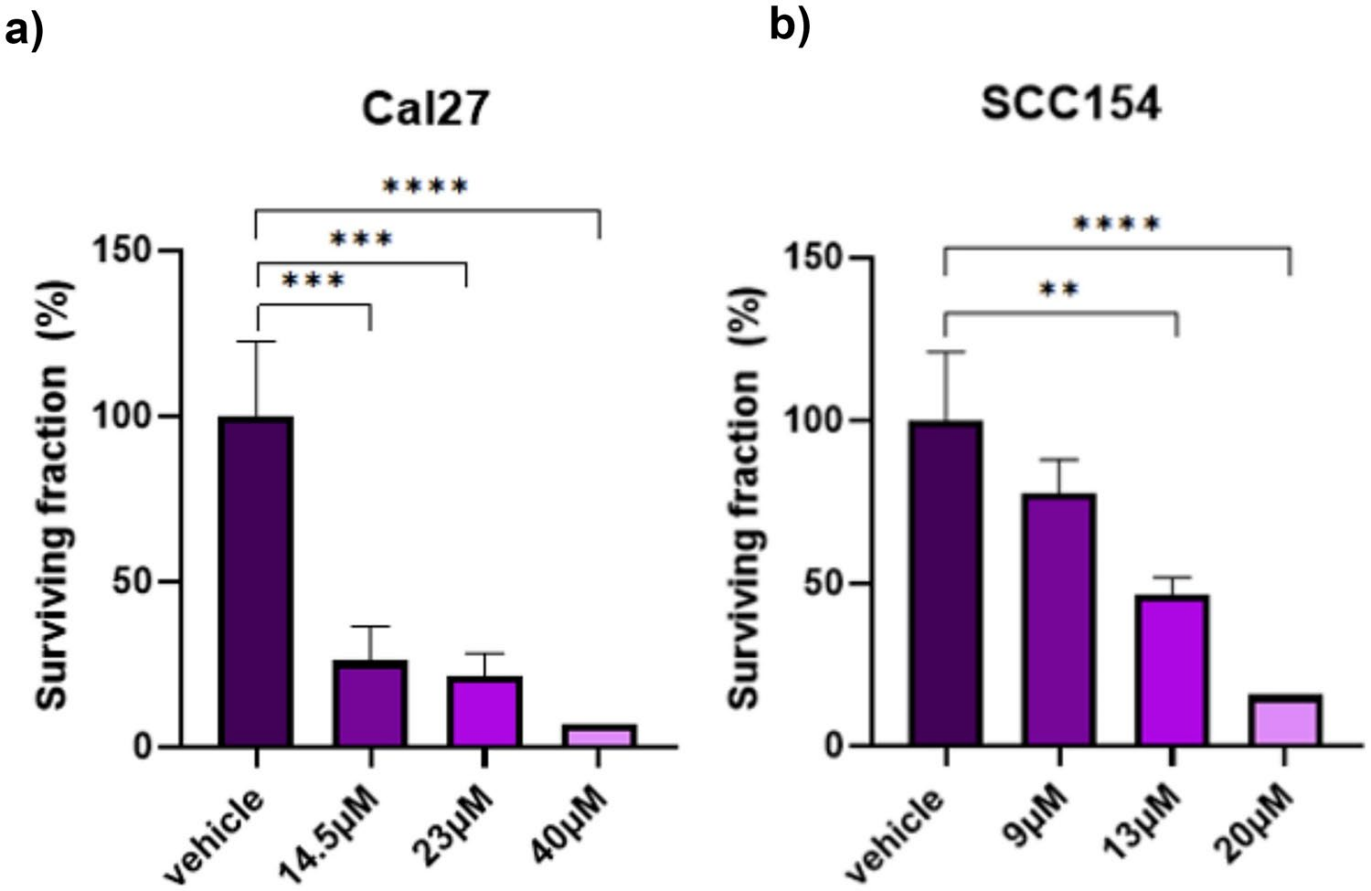
Colony-forming assay for Cal27 (**a**) and SCC154 (**b**) cell lines. Significant differences between controls and PF-treated cells are represented with asterisks (**; *p* < 0.01, ***; *p* < 0.001, ****; *p* < 0.0001)

Next, in order to assess the colony forming abilities, we performed the clonogenic assay with 1000 SCC154 cells/well and 350 cells/well for Cal27 and FaDu seeded into 12-well plates. Cal27 and SCC154 cells were treated with three PF concentrations and a DMSO vehicle control. In total, strong anti-clonogenic properties of PF were shown in both cell lines. These results are shown in Fig. 4. The surviving fraction reflects the percentage of surviving colonies normalized to the vehicle control value. PF treatment of the FaDu cell line did not deliver reproducible results, and therefore, we excluded these from the analysis. One-way ANOVA multiple comparison test showed significant differences for increasing inhibitor concentrations in both cell lines.

### PF induces apoptosis in Cal27, FaDu, and SCC154 cell lines

**Fig. 5:**
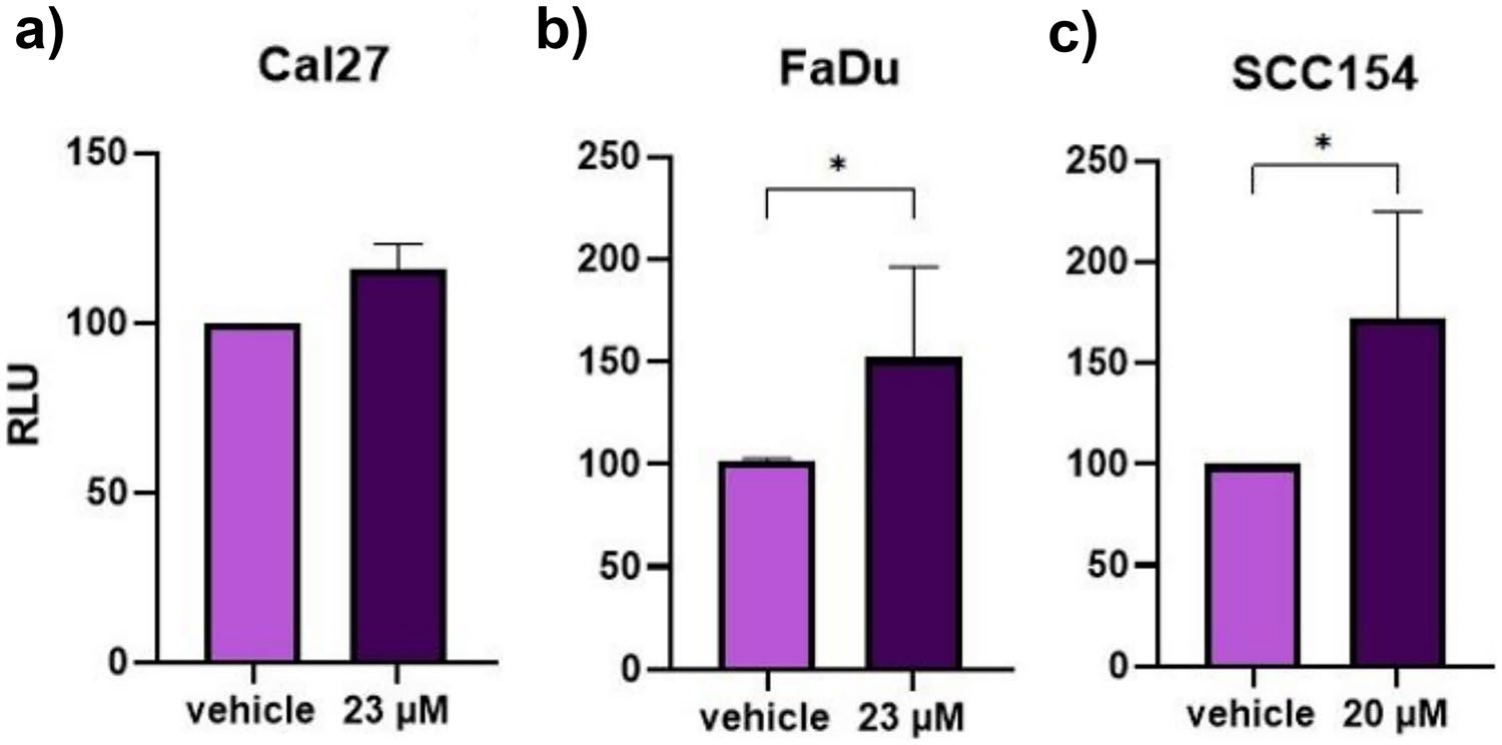
HNSCC cell lines treated with PF inhibitor show increased apoptosis. Pro-apoptotic effects of the IC50 PF concentration were revealed in all three cell lines. In particular, the effect was not significant in the Cal27 cell line (**a**), while being significant in FaDu (**b**) and SCC154 (**c**). Graphs represent mean relative light units (RLU) ± SD, normalized to DMSO control. Significant differences between controls and PF-treated cells are represented with asterisks (*; *p* < 0.05)

Finally, we assessed the pro-apoptotic potential of PF with the Caspase 3/7 Glo assay. For this, 7500 Cal27 and FaDu cells/well and 15,000 SCC154 cells/well were seeded into 96-well plates in 100 μL DMEM. All three cell lines were treated the corresponding IC50 PF concentration. Notably, the treatment induced apoptosis in all three cell lines. In particular, a significant increase was observed in FaDu and SCC154 cells. These results are depicted in Fig. 5.

## Discussion

The prognosis in patients with advanced HNSCC remained poor in recent years. Moreover, no major advancements regarding chemotherapy were made in the last decades. Furthermore, widely used cisplatin is associated with various adverse effects, often causing a transition to another agent or treatment termination. Finally, particularly treatment of HPV-positive HNSCC is set to become a major challenge in the coming decades, due to the aforementioned global rising incidence rates [2, 11–13]. Therefore, finding new treatment options and chemotherapy agents seems highly warranted.

The data on targeting the Notch pathway via GSI is sparse in HNSCC. To the best of our knowledge, only one study reported on the effects of PF in HNSCC. In particular, Zhang et al. showed that PF in combination with erlotinib suppressed cancer growth [21]. No further studies reported on anti-neoplastic properties of PF in HNSCC, particularly in HPV-positive cell lines. In the current study, we have observed anti-neoplastic effects of Notch pathway inhibition via PF in HPV-associated and HPV-unrelated HNSCC cell lines.

Generally, HPV-associated HNSCC patients have a more favorable prognosis than HPV-unrelated HNSCC. In particular, 5-year survival rates are reported as high as 80% in HPV-related HNSCC. Meanwhile, the 5-year survival rate is reported around only 50% in HPV-negative HNSCC [21]. Still, a subgroup of patients with HPV-positive HNSCC develop distant metastases and die from the disease [22, 23], which further underlines the need for the development of new treatment options particularly for this patient group. PF seems to be slightly more potent in the HPV-positive cell line and could therefore potentially contribute to improving survival in patients with high-risk HPV-positive disease.

Another broadly used treatment option in HNSCC is radiation therapy [24]. Therefore, we conducted experiments assessing the synergistic effects of radiation and PF. Importantly, we noticed combinatorial effects of PF treatment with radiation in the HPV-positive cell line. As high radiosensitivity of HPV-positive head and neck malignancies is well-known [25–28], the synergy between irradiation and treatment with PF further underlines its therapeutic potential for HPV-associated malignancies.

Besides cytotoxic effects, the anti-neoplastic potential of PF was further demonstrated in a migration assay. In particular, treatment with PF significantly decreased the gap closure in all cell lines. These are, to the best of our knowledge, first insights into the anti-migratory potential of PF in HNSCC. With regards to the anti-clonogenic effects of PF, its IC50 concentration seemed sufficient to disrupt colony formation in SCC154. Correspondingly, treatment with PF had a similar anti-clonogenic potential in the Cal27 cell line. Disrupting the clonogenic abilities via Notch pathway inhibition has been observed in other studies as well. For example, Lan et al. showed adverse effects on cell growth and migration by Notch inhibition in embryonic microenvironment [29]. Furthermore, in a study by Farah E. et al., Notch inhibition decreased in-vitro migration and colony-forming capabilities of prostate cancer [30].

Lastly, we provided evidence on the pro-apoptotic properties of PF in HNSCC in vitro. In particular, slightly stronger pro-apoptotic effects were shown in the HPV-positive cell line. Certainly, the mechanistic background underlining our findings remains unclear and warrants further investigations. Interestingly, HPV infection has shown to have anti-apoptotic properties [31] and therefore, findings regarding pro-apoptotic effects in the HPV-positive cell line require further deciphering. Similar results regarding the pro-apoptotic effects of Notch repression could be identified in the literature. In particular, Notch pathway inhibition led to an increase in apoptosis in the study by Ye et al. They observed induced apoptosis via silencing the Notch pathway in prostate cancer cells [32]. Specifically for HNSCC, Dai et al. showed that downregulation of Notch1 induced apoptosis and inhibited cell proliferation and metastasis in laryngeal cancer [33].

In total, our study provided novel results and we gained insights into the therapeutic potential of GSI in HNSCC. However, certain limitations need to be addressed. First, we conducted all experiments solely in 2D cell cultures and these do not completely reflect the native tumor microenvironment of HNSCC patient tumors. Furthermore, the mechanistic background behind the antitumor effects of PF was not analyzed and these could be deciphered with molecular biology experiments such as Western blotting. In particular, the direct downregulation of the Notch pathway by the GSI was not assessed. Most importantly, we assessed PF antitumor properties only in one HPV-positive cell line. Therefore, in order to be able to make definitive statements on PF effects in HPV-positive HNSCC, our findings need to be validated in further HPV-dependent cell lines.

## Data Availability

The datasets generated during and/or analysed during the current study are available from the corresponding author on reasonable request.
